# DNA methylation and miRNA-1296 act in concert to mediate spatiotemporal expression of KPNA7 during bovine oocyte and early embryonic development

**DOI:** 10.1186/s12861-019-0204-x

**Published:** 2019-12-02

**Authors:** Lei Wang, Jacqelyn M. Hand, Liyuan Fu, George W. Smith, Jianbo Yao

**Affiliations:** 1 Laboratory of Animal Biotechnology and Genomics, Division of Animal and Nutritional Sciences, West Virginia University, Morgantown, WV 26506 USA; 2 Laboratory of Mammalian Reproductive Biology and Genomics, Departments of Animal Science and Physiology, Michigan State University, East Lansing, MI 48824 USA

**Keywords:** DNA methylation, miRNA, Oocyte, Early embryonic development, Maternal, Cattle

## Abstract

**Background:**

Epigenetic regulation of oocyte-specific maternal factors is essential for oocyte and early embryonic development. KPNA7 is an oocyte-specific maternal factor, which controls transportation of nuclear proteins important for early embryonic development. To elucidate the epigenetic mechanisms involved in the controlled expression of KPNA7, both DNA methylation associated transcriptional silencing and microRNA (miRNA)-mediated mRNA degradation of *KPNA7* were examined.

**Results:**

Comparison of DNA methylation profiles at the proximal promoter of *KPNA7* gene between oocyte and 6 different somatic tissues identified 3 oocyte-specific differentially methylated CpG sites. Expression of *KPNA7* mRNA was reintroduced in bovine kidney-derived CCL2 cells after treatment with the methylation inhibitor, 5-aza-2′-deoxycytidine (5-Aza-CdR). Analysis of the promoter region of *KPNA7* gene in CCL2 cells treated with 5-Aza-CdR showed a lighter methylation rate in all the CpG sites. Bioinformatic analysis predicted 4 miRNA-1296 binding sites in the coding region of *KPNA7* mRNA. Ectopic co-expression of miRNA-1296 and KPNA7 in HEK293 cells led to reduced expression of KPNA7 protein. Quantitative real time PCR (RT-qPCR) analysis revealed that miRNA-1296 is expressed in oocytes and early stage embryos, and the expression reaches a peak level in 8-cell stage embryos, coincident with the time of embryonic genome activation and the start of declining of KPNA7 expression.

**Conclusions:**

These results suggest that DNA methylation may account for oocyte-specific expression of KPNA7, and miRNA-1296 targeting the coding region of *KPNA7* is a potential mechanism for *KPNA7* transcript degradation during the maternal-to-zygotic transition.

## Background

Successful germ cell development and differentiation during oogenesis and early embryogenesis is accomplished through the help of nuclear proteins such as transcription factors and chromatin-remodeling factors that act in the nucleus [1]. A family of nuclear transporters called karyopherins are the major players in the translocation of nuclear proteins through an active, energy-dependent nuclear import system. To date, seven members of karyopherin alpha (KPNA) have been identified in mammals; only *KPNA7* is strictly expressed in oocytes and early embryos [2–4]. In mice, *KPNA7* knockout lead to fetal lethality, sex imbalance and abnormalities of epigenetic modifications (e.g. down-regulation of histone H3K27me3) [3]. In livestock species, such as cattle and pigs, knockdown of KPNA7 significantly reduces blastocyst rate through inducing arrested embryonic development [2, 4]. In cattle, the expression of KPNA7 is high in germinal vesicle (GV) oocytes through 8-cell stage embryos but drops to barely detectable levels in morula and blastocyst stage embryos [2]. The sudden drop of mRNA levels during the 8–16 cell stages is coincident with the time of maternal-to-zygotic transition (MZT) in cattle. To date, little is known about the mechanistic control of tissue- and stage-specific expression of KPNA7.

DNA methylation at the 5-position of cytosine (5mC) largely occurs at CpG dinucleotides and is required for normal gametogenesis and embryogenesis in mammals [5]. In the early stages of oogenesis, the genome of embryonic germ cells is dynamically reprogrammed during cell differentiation and the differentially methylated regions begin to maintain the monoallelic expression of imprinted genes [6–8]. Genes of developmental importance, such as germ cell-specific factors Nanog, Dazl, Pou5f1 and Sry, which control primordial germ cell development, are all regulated through DNA methylation-mediated mechanisms [9–11]. Tissue-specific and differentially methylated regions are common in the mammalian genome and correspond to different cell types in an organism [12]. Since DNA methylation profile is tissue-specific, it is reasonable to believe that DNA methylation, particularly, methylation in the CpG sites located in the proximal promoter surrounding the transcription start site (TSS), plays a role in controlling the expression of oocyte-specific maternal factors.

Maternal effect genes are the major driving force to facilitate oocyte maturation, fertilization and embryonic genome activation [13]. However, after MZT, almost 90% of the maternal transcripts are degraded and the clearance of maternal transcripts is proved to be essential for normal embryonic development [14]. For example, in *Xenopus laevis*, abundance of the oocyte-specific maternal transcript *c-mos* is reduced quickly after fertilization, and introducing c-mos protein into 2-cell stage embryo led to development block [15]. This phenomenon was observed in the mouse and other species, which indicates that maternal transcript degradation is required for normal embryonic development [16]. Multiple negative regulatory mechanisms including mRNA deadenylation, interaction with RNA-binding proteins and miRNA-mediated degradation are involved in post-transcriptional degradation of maternal transcripts [17]. miRNAs such as miRNA-430 in zebrafish and miRNA-427 in *Xenopus* were shown to be present prior to embryonic genome activation and further studies revealed more evidence to support the role of these miRNAs in degradation of hundreds of maternal transcripts [18–20]. In cattle, a number of oocyte-specific maternal transcripts (e.g. *NPM2*, *NOBOX* and *FIGLA*) were shown to be targeted by miRNAs for their degradation [21–23].

In the present study, we identified oocyte-specific differentially methylated CpG sites in the promoter region of bovine *KPNA7* gene and demonstrated that *KPNA7* mRNA is potentially targeted by miRNA-1296 for degradation. The results suggest distinctive controlling mechanisms for tissue- and stage-specific expression of bovine *KPNA7* gene during oocyte and early embryonic development.

## Results

### *KPNA7* promoter is differentially methylated in bovine oocyte and somatic tissues

**Fig. 1:**
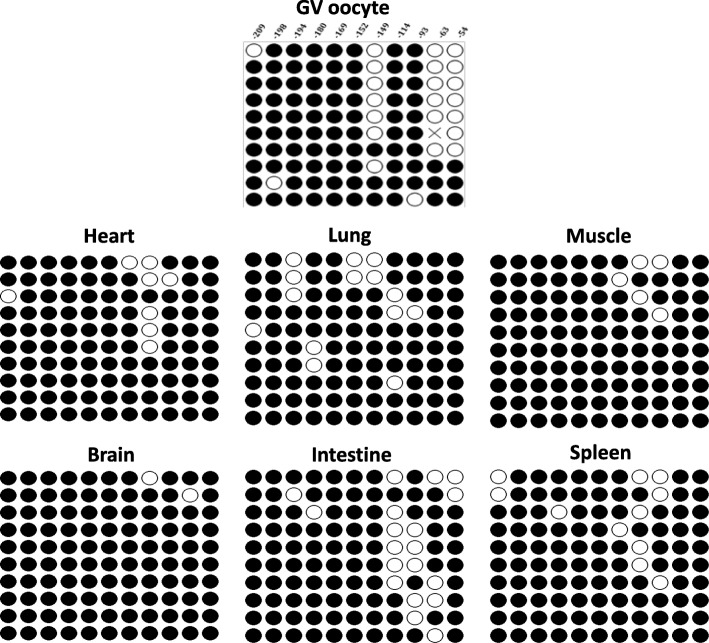
Methylation status of 11 CpG sites in the proximal promoter of bovine *KPNA7* gene in GV oocytes and 6 somatic tissues. Three tissue-specific differentially methylated sites (− 149, − 63 and − 54) were detected. Close circles indicate methylated sites; open circles indicate demethylated sites

It has been generally believed that DNA hypermethylation at the proximal promoter can repress gene transcription by interfering with transcription initiation [24]. Therefore, differentially methylated promoter region of *KPNA7* gene may contribute to its oocyte-specific expression. Analysis of the genomic DNA sequence around the transcription start site (TSS) of *KPNA7* gene using an online algorithm (Methprimer) revealed multiple CpG sites including a CpG island containing 8 CpG sites (− 221 to − 114) located upstream of the TSS (Additional file 1. Figure S1). Using bisulfite conversion and subsequent DNA sequencing, the methylation status of 11 consecutive CpG sites including 8 within the CpG island and 3 located right before the TSS at the CpG island shore region were characterized. Three hypomethylated CpG sites (− 149, − 63 and − 54) with high demethylation rates (60–70%) were identified in the GV oocyte in comparison to 6 different somatic tissues including heart, lung, muscle, brain, intestine and spleen (Fig. 1). The hypomethylation pattern observed in the 3 sites was found to exist only in the GV oocyte with no more than 20% demethylation observed in any of the somatic tissues examined, which indicates a tissue-specific methylation pattern of these 3 CpG sites.

### Treatment with 5-Aza-CdR reintroduces expression of KPNA7 in CCL-22 cells

**Fig. 2:**
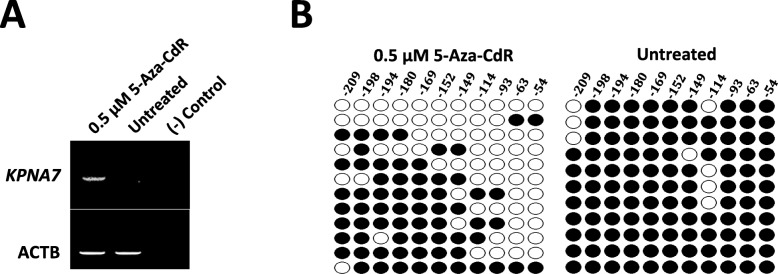
5-Aza-CdR treatment of CCL-22 cells reintroduces KPNA7 expression by demethylation of CpG sites in the proximal promoter of bovine *KPNA7* gene. **a** Expression of *KPNA7* was detected in 5-Aza-CdR treated CCL-22 cells by RT-PCR. **b** Methylation status of 11 CpG sites in the proximal promoter of bovine *KPNA7* gene in the 5-Aza-CdR treated and control CCL-22 cells. Demethylation of the CpG sites in the treatment group, and hypermethylation of the CpG sites in the control group were observed. Three oocyte-specific hypomethylated sites (− 149, − 63 and − 54) are all methylated in the control group. Close circles indicate methylated sites; open circles indicate demethylated sites

CCL-22 cells are derived from bovine kidney cells, which do not express KPNA7 naturally. 5-Aza-CdR inhibits activity of DNA methyltransferase 1 (DNMT1), an enzyme that maintains the status of DNA methylation. As cells divide, treatment of 5-Aza-CdR results in global demethylation of the cell genome, and thereby induces the expression of genes silenced by DNA methylation [25]. As shown in Fig. 2a, *KPNA7* expression was induced in the 5-Aza-CdR treated cells. Bisulfite sequencing of CCL-22 cells from the treatment (0.5 μM) and the control groups showed demethylation of the *KPNA7* proximal promoter in the treatment group, whereas in the control group, hypermethylation was observed (Fig. 2b), which agrees with the RT-PCR results showing no expression of *KPNA7* mRNA in the control group. Three oocyte-specific hypomethylated sites (− 149, − 63, and − 54) are all methylated in the control group, and in the treatment group they underwent partial demethylation showing resemblance to the methylation status found in oocytes. These results indicate that DNA methylation in the proximal promoter of *KPNA7* gene is critical for its tissue-specific expression, and the 3 oocyte-specific hypomethylated sites may play a primary role in controlling *KPNA7* tissue-specificity.

### Promoter demethylation does not activate *KPNA7* expression after embryonic genome activation

**Fig. 3:**
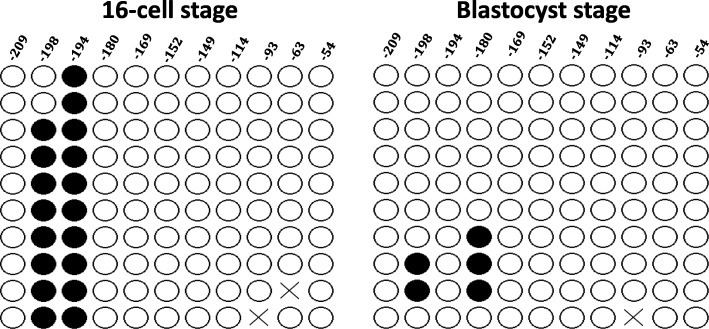
Methylation status of 11 CpG sites in the proximal promoter of bovine *KPNA7* gene in 16-cell and blastocyst stage embryos. Hypomethylation of the CpG sites were observed in both stage embryos. Close circles indicate methylated sites; open circles indicate demethylated sites

As a maternal effect gene, *KPNA7* is only expressed in oocyte and early embryos in which the expression level is high until it rapidly decreases after MZT (~ 16-cell stage) and does not increase again until the formation of primordial germ cells in late pregnancy [2]. Therefore, 16-cell and blastocyst stage embryos were used to test if silencing of *KPNA7* expression was due to DNA methylation in the promoter region. Bisulfite sequencing and subsequent analysis showed that in 16-cell stage embryos, the *KPNA7* proximal promoter is heavily hypomethylated (Fig. 3). At the further developmental stage, when the embryonic genome is completely activated, the *KPNA7* proximal promoter is still extremely hypomethylated. This pattern shows that DNA methylation of the CpG sites at the proximal promoter does not control *KPNA7* expression in embryos after MZT, and thus silencing of *KPNA7* expression in embryos after the 16-cell stage is likely due to other mechanisms.

### miRNA-1296 is potentially involved in translational silencing of bovine KPNA7

**Fig. 4:**
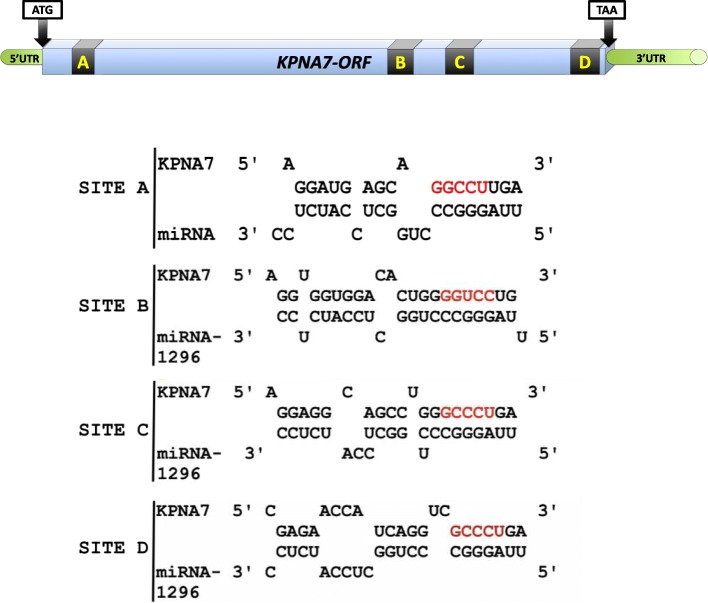
Prediction of miRNA-1296 binding sites in the coding region of bovine *KPNA7* mRNA. Four putative miRNA-1296 binding sites (A, B, C and D) were predicted using the online software Microinspector (http://bioinfo.uni-plovdiv.bg/microinspector/)

**Fig. 5:**
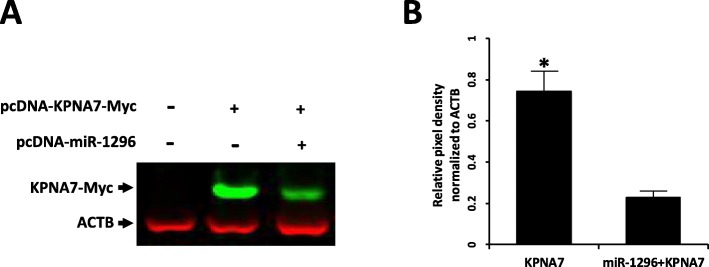
miRNA-1296 suppresses the expression of KPNA7 protein in HEK293 cells. **a** A representative western blot showing reduction of KPNA7 protein in HEK293 cells expressing miRNA-1296. ACTB was used as a loading control. The experiment was repeated three times. **b** ImageJ software was used to quantify the protein bands. Abundance of KPNA7 protein was normalized relative to abundance of ACTB protein. Data are expressed as mean relative pixel density (*n* = 3, mean ± SEM). Asterisk indicates statistical difference (*P* < 0.05)

**Fig. 6:**
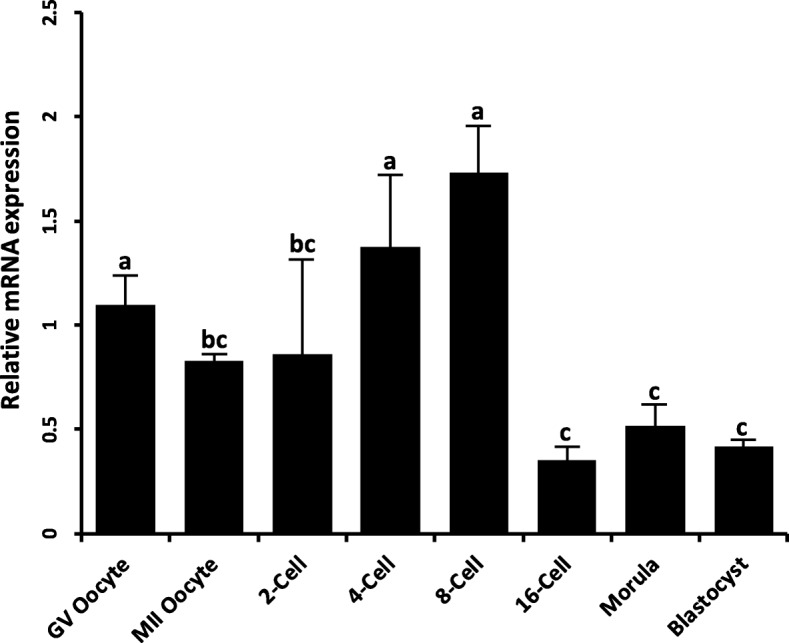
Relative abundance of miR-1296 mRNA in bovine oocytes and in vitro produced bovine early embryos (*n* = 4 pools of five oocytes/embryos each). Quantity of miRNA was normalized relative to abundance of miRNA-125b. Different letters indicate statistical difference (P < 0.05)

To identify miRNAs that may target KPNA*7*, the *KPNA7* cDNA sequence (GenBank accession No. FJ754641) was uploaded to the online algorithm “Microinspector” (http://bioinfo.uni-plovdiv.bg/microinspector/) to predict miRNA binding sites. Four miRNA-1296 recognition elements in the coding region of *KPNA7* mRNA were predicted (Fig. 4). Co-transfection of expression constructs harboring miRNA-1296 and KPNA7 showed a reduction of KPNA7 protein expression in HEK293 cells compared to the cells transfected with KPNA7 expression construct alone (Fig. 5a). This experiment was repeated three times and quantitative analysis of western blot data showed a significant reduction of KPNA7 protein in the co-transfection group (Fig. 5b). The results suggest that miRNA-1296 may play a role in the regulation of bovine KPNA7 expression at the post-transcriptional level. RT-qPCR analysis showed that miRNA-1296 expression reaches its peak level in the 8-cell stage embryo, which is coincident with the start of declining of KPNA7 expression (Fig. 6). The data supports our hypothesis that miRNA-1296 acts as a functional physiological regulator for KPNA7 expression during early embryogenesis.

## Discussion

Tissue-specific deferential methylation has become an important aspect in epigenetic-related gene expression studies [26, 27]. Comparisons between oocyte and sperm, developmental germ cell stages, and different embryonic stages showed stage- or germ cell- specific differentially methylated regions [28, 29]. In this study, we demonstrated that methylation of 3 CpG sites (− 149, − 63 and − 54) in the promoter region controls tissue-specific expression of bovine *KPNA7* gene. Two of the differentially methylated sites are located in the CpG island shore region (− 63 and − 54). The role of CpG island shore regions in controlling gene expression has been recently demonstrated [30, 31].

Control of gene expression by specific CpG sites was observed in a previous study in prostate cancer cells [32]. When treated with a minimal concentration of 5-Aza-CdR, one CpG site was hypersensitive to the challenge and methylation of this single site led to silencing of *PMP24* gene. In the present study, we observed that demethylation of CpG sites tends to spread from the TSS towards the upstream positions. Two sites (− 63 and − 54) closest to the TSS had the highest demethylation level than did the sites located upstream, suggesting a primary role of these 2 sites in controlling the expression of *KPNA7* gene.

After fertilization, the paternal genome undergoes spontaneous global demethylation before first cleavage but the maternal genome retains its methylation status until the first cleavage event in which demethylation occurs passively with each cell division [33]. In cattle, the embryonic genome is mostly activated by the 16-cell stage and is completely activated in the blastocyst stage embryo. The methylation status of the 5′ terminal region near the TSS of developmentally important genes has been surveyed and all the genes, including *Oct4*, *Sox2*, *Nanog*, *Rex1* and *Fgf4*, experienced demethylation after fertilization and the expression of these genes were not accompanied by the demethylation of their promoter regions [34]. Therefore, the dynamic methylation changes in early embryonic development, especially during MZT, might not contribute to activation of gene transcription. In this case, the reduction of *KPNA7* mRNA level is explained by inactivation of transcriptional activity, however, on the other hand, rapid mRNA degradation might also be a reason for diminishment of the *KPNA7* transcript.

With 4 predicted miRNA-1296 binding sites in the coding region of *KPNA7* mRNA, the functional role of miRNA-1296 in regulation of KPNA7 expression was investigated. Our co-transfection experiments indicated that miRNA-1296 may play a role in down-regulating the expression of KPNA7 protein. However, this finding is limited as we did not show specific binding of miRNA-1296 to the predicted binding sites on *KPNA7* mRNA. Further experiments using reporter constructs with mutated miRNA-1296 binding sites are needed to test whether the effect of miRNA-1296 on KPNA expression is direct or indirect.

The classic miRNA-mediated post-transcriptional regulation of mRNAs has been believed to be confined to the 3’UTR region of the transcripts [35]. However, new studies have demonstrated the existence of many naturally occurring alternative binding regions of miRNA in mammalian cells [36, 37]. During embryogenesis, miR-134, miR-296 and miR-470 target the coding regions of transcription factors Nanog, Oct4 and Sox2 in various combinations, leading to transcriptional and morphological changes in mouse embryonic stem cells [36]. In bovine species, Nanog, Oct4 and Sox2 are all oocyte-specific maternal factors that govern early embryonic development by regulating the pluripotency of blastomeres [38, 39]. Thus, miRNA targeting the coding regions of developmentally important factors might be a characteristic feature in post-transcriptional regulation of maternal effect factors.

Targets of miRNA-1296 have been studied and shown to be highly involved in essential DNA replication [40]. miRNA-1296 targets *MCM2* mRNA in prostate cancer cells and over expression of miRNA-1296 results in a significant decrease in *MCM2* mRNA, protein, and S-phase of the cell cycle. MCM2 is an essential DNA replication factor that is highly expressed in cancer cells as well as in the oocyte and is highly involved in resumption of meiosis in mammalian oocytes [41]. The potential involvement of miRNA-1296 in regulating the expression of KPNA7 supports a new role of this miRNA in the control of oocyte and early embryonic development.

Studies in human cancers have shown that miRNAs and DNA methylation can mutually regulate each other [42]. For example, the miRNA-29 family regulates DNA methylation by targeting DNA methyltransferases (DNMT3a and DNMT3b) in lung cancer tissues [43], and the expression of the miRNA-34 family members (miRNA-34b and miRNA-34c) is silenced by hypermethylation of the promoters of the miRNA genes in gastric cancer cells [44]. It is not clear how the expression of bovine miRNA-1296 is regulated during embryogenesis but DNA methylation of the miRNA gene promoter could play a role in controlling its expression. There is currently no evidence showing the involvement of miRNA-1296 in modulating DNA methyltransferases, thus affecting DNA methylation.

## Conclusions

In this study, we found that the oocyte restricted expression of bovine KPNA7 is regulated by DNA methylation at the proximal promoter and demethylation of 3 CpG sites is closely related to tissue-specific expression of this gene. We also provided evidence showing that miRNA-1296 is potentially involved in translational silencing of bovine KPNA7 through binding sites in the coding region of *KPNA7* mRNA. This study combined two aspects of epigenetic regulation of gene expression and discovered distinctive controlling mechanisms for tissue- and stage-specific expression of bovine *KPNA7* gene.

## Methods

### Sample collection

Bovine tissue samples including heart, lung, muscle, brain, intestine and spleen were collected at a local abattoir. Bovine GV oocytes and early stage embryos were purchased from Bomed, Inc. (Madison, WI). All samples were frozen in liquid nitrogen and stored at − 80 °C until use.

### Plasmid construction

The open reading frame (ORF) of bovine *KPNA7* cDNA was PCR amplified from a *KPNA7* expression plasmid [2] and cloned into pcDNA3.1/myc-His vector (Invitrogen, Carlsbad, CA) using a forward primer containing a Kozak sequence and BamHI site and a reverse primer containing a XhoI site (Additional file 2, Table S1). The plasmid designed to express the bovine miRNA-1296 was prepared by PCR amplification of a 291 bp genomic fragment containing the pre-miRNA-1296 followed by cloning into pcDNA3.1 vector using a forward primer containing a BamHI site and a reverse primer containing a PmeI site (Additional file 2, Table S1). Both constructs were sequenced to ensure that no mutations were introduced during PCR amplification.

### Bisulfite sequencing

DNA samples isolated from oocytes/embryos or somatic tissues were treated by bisulfite following the manufacturer’s instructions of the EZ DNA Methylation-Direct™ kit (Zymo Research, Irvine, CA). Primers (Additional file 2, Table S1) were designed using Methprimer online program and were used in a 25-μl PCR reaction for the first round of 40 cycles. Cycling conditions were as follows: 95 °C for 9 min followed by 40 cycles of 95 °C for 30 s, 54 °C for 30 s, 72 °C for 30 s and a final extension of 5 min at 72 °C. Nested PCR of 35 cycles was performed using the PCR product as a template in a higher annealing temperature at 58 °C and a shorter denaturation time of 5 min at 95 °C. The DNA regions of CpG sites were amplified, and three independent PCR reactions were performed. The PCR products were purified, pooled together and cloned into pGEM-T Easy vector (Promega). After verification, 10 clones for each DNA sample were sequenced, and the sequences were analyzed using the online tool *QUMA* (http://quma.cdb.riken.jp/).

### Cell culture

HEK293 cells were cultured in DMEM (Invitrogen, Carlsbad, CA) containing 10% FBS. For transient transfection, Xtremegene 9 (Roche Applied Science, Indianapolis, IN) was used according to manufacturer’s instructions. Following transfection, cells were incubated for 24 h before harvest for western blot analysis. CCL22 cells were cultured in DMEM containing 10% HS. 5-Aza-CdR was added to the culture medium with a final concentration of 0.5 μM in 6-well plates (Corning Inc., Corning, NY) seeded with cells 24 h before the treatment. Treatment continued for 3 days with fresh 5-Aza-CdR changed every 24 h. After 3 days, cells were harvested and stored at − 80 °C until use.

### Western blot analysis

Electrophoresis and transfer were performed according to a previous study with minor modifications [45]. HEK293 cell were harvested with Pierce IP Lysis Buffer (Thermo Fisher Scientific, Waltham, MA), and 10 μl of cell lysate were mixed with an equal volume of Laemmli sample buffer (Bio-Rad Laboratories, Hercules, CA). Protein samples (15 μg/each) were separated on a 4–20% gradient ready gel (Bio-Rad) and transferred onto a Immobilon-FL PVDF membrane (Millipore, Billerica, MA). Following transfer and blocking in 5% nonfat dry milk in PBS containing 0.1% Tween-20 (PBST) for one hour, the membrane was incubated in anti-Myc antibody (Sigma-Aldrich, St. Louis, MO) diluted 1:1000 and anti-ACTB antibody (GenScript, Piscataway, NJ) diluted 1:1000 in blocking buffer overnight at 4 °C. After 3 washes, two secondary antibodies IRDye 800CW goat anti-rabbit and IRDye 680LT goat anti-mouse (Li-COR, Lincoln, NE) were added to the blocking buffer and incubated for 20 min. Detection of protein was performed following the instructions of the Odyssey system (Li-COR, Lincoln, NE). ImageJ software was used to quantify the protein bands. Abundance of KPNA7 protein was normalized relative to the abundance of ACTB protein.

### Quantitative real-time PCR

cDNA from oocyte and embryo samples (*n* = 3 pools of five each) was prepared by lysing the samples in 1× miScript RT buffer containing 0.5% NP-40 at 95 °C for 5 min followed by addition of miScript reverse transcriptase mix (Qiagen, Valencia, CA) and incubation at 37 °C for 60 min. The cDNA was then used for determination of relative amount of miR-1296 by RT-qPCR using the miRNA-1296 specific primer and the miScript universal primer (Qiagen, Valencia, CA). Bovine miRNA-125b was used as an endogenous control as this miRNA is expressed consistently in preimplantation embryos [46]. RT-qPCR analysis was performed on the Bio-Rad CFX96 system. The iQ™ SYBR Green Supermix (Bio-Rad, Hercules, CA) was used in 20 μl reaction volumes containing 100 nM of each primer and 5 μl of diluted cDNA. Cycling parameters were 95 °C for 15 min, and then 40 cycles of 95 °C for 15 s, 55 °C for 30 s and 70 °C for 30 s. Standard curves for the target and control miRNA were constructed using 10-fold serial dilution of a pooled cDNA sample.

## Supplementary information

**Additional file 1: Figure S1.** Identification of CpG sites in the proximal promoter of bovine *KPNA7* gene. Analysis of DNA sequence around the transcription start site (TSS) of bovine *KPNA7* gene using Methprimer (http://www.urogene.org/cgi-bin/methprimer2/MethPrimer.cgi) revealed multiple CpG sites including a CpG island containing 8 CpG sites (− 221 to − 114) located upstream of the TSS. (PPTX 69 kb)

**Additional file 2: Table S1.** List of primers used in this study. (XLSX 9 kb)

## Data Availability

The datasets used and/or analyzed during the current study are available from the corresponding author on reasonable request.

## Abbreviations

5-Aza-CdR: 5-aza-2′-deoxycytidine
5mC: 5-position of cytosine
DNMT1: DNA methyltransferase 1
GV: germinal vesicle
KPNA: karyopherin alpha
miRNA: microRNA
MZT: maternal-to-zygotic transition
RT-qPCR: quantitative real time PCR
TSS: transcription start site
