# Supplementary figures and images for: DNA methylation and miRNA-1296 act in concert to mediate spatiotemporal expression of KPNA7 during bovine oocyte and early embryonic development
Source: BMC Dev Biol. 2019 Dec 2;19:23. doi: 10.1186/s12861-019-0204-x (PMC6886206; doi:10.1186/s12861-019-0204-x)

## Slide 1
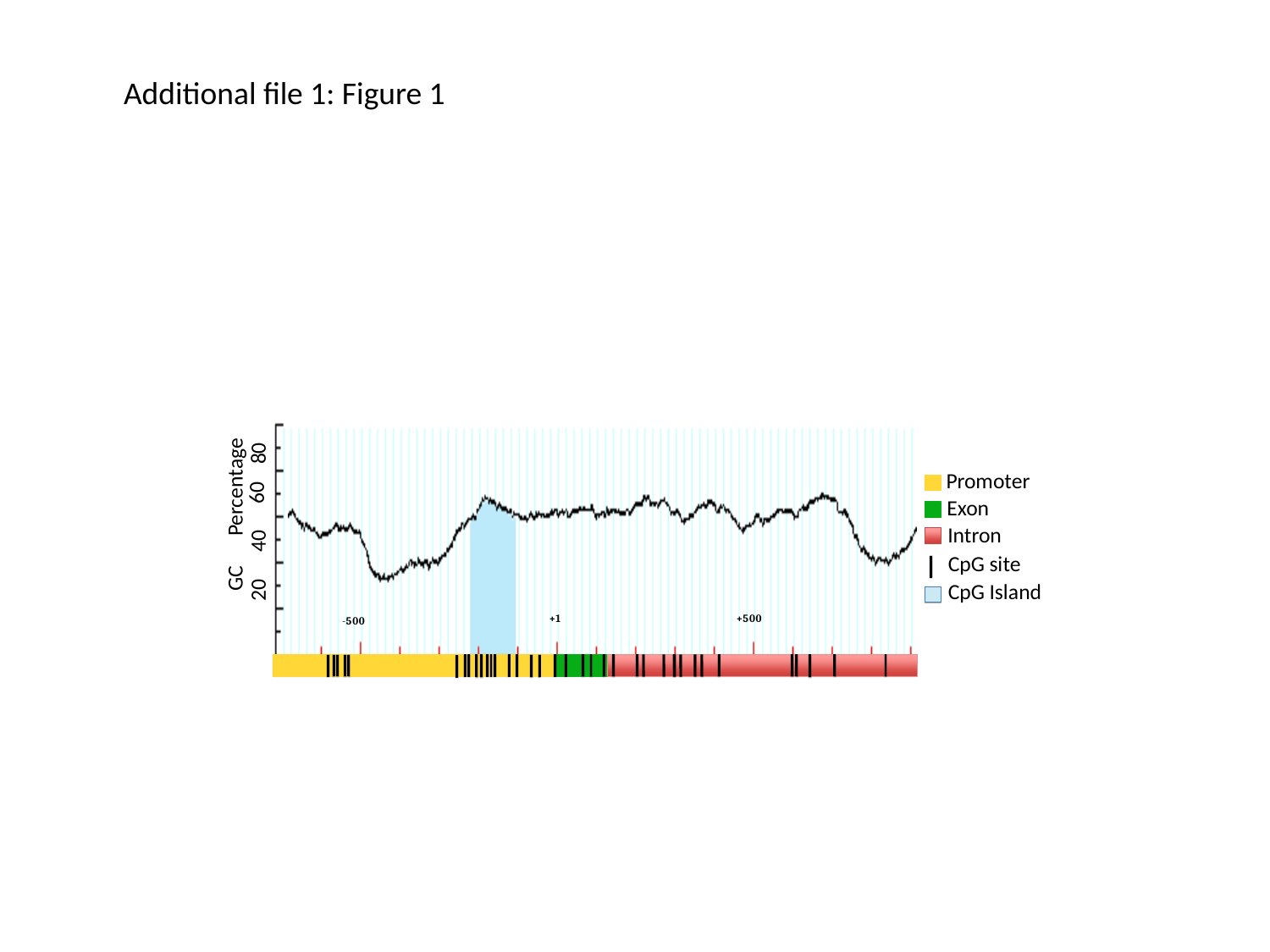

Additional file 1: Figure 1

Supplement: Supplementary file 1 — Additional file 1: Figure S1. Identification of CpG sites in the proximal promoter of bovine KPNA7 gene. Analysis of DNA sequence around the transcription start site (TSS) of bovine KPNA7 gene using Methprimer (http://www.urogene.org/cgi-bin/methprimer2/MethPrimer.cgi) revealed multiple CpG sites including a CpG island containing 8 CpG sites (− 221 to − 114) located upstream of the TSS. (PPTX 69 kb) [file 12861_2019_204_MOESM1_ESM.pptx]
